# Balancing Act: Acute and Contextual Vestibular Sensations of Cranial Electrotherapy Stimulation Using Survey and Sensor Outcomes in a Non-Clinical Sample

**DOI:** 10.3390/brainsci14010087

**Published:** 2024-01-17

**Authors:** Kayla S. Sansevere, Joel A. MacVicar, Daniel R. Samuels, Audrey K. Yang, Sara K. Johnson, Tad T. Brunyé, Nathan Ward

**Affiliations:** 1 Department of Psychology, Tufts University, 490 Boston Ave., Medford, MA 02155, USAnathan.ward@tufts.edu (N.W.); 2 Eliot-Pearson Department of Child Study and Human Development, Tufts University, 105 College Ave., Medford, MA 02145, USA; 3 U.S. Army Combat Capabilities Development Command Soldier Center, 15 General Greene, Natick, MA 01760, USA; 4 Center for Applied Brain and Cognitive Sciences, 200 Boston Ave., Suite 1800, Medford, MA 02155, USA

**Keywords:** non-invasive brain stimulation, neuromodulation, side effects, vestibular sensations, cognitive demand, physical demand, postural sway, Alpha-Stim

## Abstract

Cranial electrotherapy stimulation (CES) delivers low-intensity electrical currents to the brain to treat anxiety, depression, and pain. Though CES is considered safe and cost-effective, little is known about side effects emerging across different contexts. Our objective was to investigate how varying physical and cognitive demands impact the frequency and intensity of CES vestibular sensations in a sample of healthy young adults. We used a 2 (stimulation: sham, active) × 2 (physical demand: static sway, dynamic sit-to-stand) × 2 (cognitive demand: single-task remain silent, dual-task count backward) repeated measures design. Vestibular sensations were measured with surveys and wearable sensors capturing balance changes. Active stimulation did not influence reported vestibular sensations. Instead, high physical demand predicted more sensation reports. High cognitive demand, but not active stimulation, predicted postural sway unsteadiness. Significant effects of active stimulation on balance were observed only during the dynamic sit-to-stand transitions. In summary, CES induces vestibular sensations only for a specific outcome under certain circumstances. Our findings imply that consumers can safely maximize the benefits of CES while ensuring they are taking steps to minimize any potential side effects by considering their context and circumstances.

## 1. Introduction

Cranial electrotherapy stimulation (CES) is a neuromodulation method involving the indirect administration of brain stimulation via low-intensity pulsing electrical currents to the central nervous system, most commonly via electrodes attached to the earlobes [1,2,3,4,5,6]. Over the past few decades, CES has become increasingly popular for treating anxiety, depression, and pain because it is user friendly, safe, and affordable [1,2,3,4,5,6]. Users may apply CES when there are little to no demands, like sitting on a couch in a quiet room. Individuals might also use CES during physically and cognitively demanding situations, such as navigating an unfamiliar environment wearing a heavy rucksack [7]. Despite the wide range of applications in use, little is known about the impacts of CES under different circumstances, especially concerning side effects. Because side effects are more likely to emerge under specific circumstances, such as when physical and cognitive demands are high, it is critical to investigate the safety and efficacy of CES in diverse contexts using complementary methods.

Although the exact mechanistic underpinnings of CES are unknown, several putative explanations exist to elucidate the effects of CES on the brain, cognition, and behavior. Computational modeling research shows that electrical currents can pass through the skull to reach cortical and subcortical regions [8,9]. When electrical currents reach subcortical regions like the thalamus, this may increase the release of neurotransmitters like gamma-aminobutyric acid (GABA) to regulate neuron excitability and maintain balanced brain activity [10]. Increased GABA release could have downstream behavioral and mood effects like reduced anxiety and depression [11]. It is also possible that electrical currents reaching the hypothalamic–pituitary–adrenal (HPA) axis could regulate cortisol levels to maintain homeostasis in prolonged, stressful situations [12,13,14,15,16,17,18,19]. Such a mechanism could explain the subjectively calming effects of CES and could be ideal for sustaining performance in the circumstances demanding higher-order executive functions and homeostasis. However, of the handful of studies investigating the effects of CES on neurotransmitters and hormones, most have found no reliable physiological effects despite significant effects on subjective self-reported mood states [20,21,22].

One of the most commonly reported side effects of CES is vestibular problems like dizziness and unsteadiness [23,24,25]. The precise source of CES-induced vestibular side effects remains unclear. Perhaps the anatomical positioning of CES electrodes activates the otolith organs or semi-circular canals, like that found with galvanic vestibular stimulation (GVS) [26,27]. Abnormal vestibular sensations could lead to consequences like poor balance and slowed walking speed, which introduces risks in more vulnerable populations such as older adults [28]. Those engaged in high-stakes, physically and cognitively demanding occupational tasks [29,30,31] are also likelier to experience unwanted side effects [32,33,34,35,36,37].

The circumstances under which side effects, such as those mentioned above, might occur remain to be identified. Notably, there needs to be more consistency in measuring and reporting the side effects of CES devices. Most published papers do not assess or report vestibular or cutaneous sensation differences across active and sham conditions [21,38,39,40,41,42,43]. Furthermore, when researchers do ask participants about side effects, it is typically at the end of the protocol when it is more difficult to reliably attribute side effects (or a lack thereof) to CES.

Our objective was to examine how varying physical and cognitive demands influence the frequency and intensity of CES vestibular sensations assessed via surveys and wearable sensors. Our objective was narrowed to a non-clinical sample of healthy young adults. We made four primary hypotheses. First, participants would report more vestibular sensations when they receive active rather than sham stimulation; such sensations may or may not be associated with behavioral changes in balance. Second, we hypothesized more reports of vestibular sensations when physical demand was highly dynamic than static. Third, we hypothesized that relatively high cognitive demand would increase reports of vestibular sensations and the observable frequency of balance perturbations. Finally, we hypothesized potential interactions wherein active CES compared to sham will exacerbate both survey and sensor vestibular effects when physical and cognitive demands are high.

## 2. Method

### 2.1. Design

We used a 2 (stimulation: sham, active) × 2 (physical demand: static sway, dynamic sit-to-stand) × 2 (cognitive demand: low single-task, high dual-task) repeated measures design. These eight conditions resulted in eight trials. For trials 1–4, participants received either sham or active CES; the cable was switched to either active or sham CES for trials 5–8. In every two trials, participants alternated between the sway position and sit-to-stand (STS) transitions; the relevant balance outcomes were captured for each trial. In every trial, cognitive demand was manipulated where participants remained silent for the low-cognitive demand (i.e., single-task) condition and performed the serial subtraction task for the high-cognitive demand (i.e., dual-task) condition. Participants reported their physical sensations after every trial. The experiment was a single session. With our counterbalanced design, trials were presented in 64 unique iterations; this determined our sample size.

### 2.2. Participants

Participants (*N* = 64) were recruited via the Tufts University Department of Psychology Paid Research Participation System hosted by Sona Systems. On average, participants were 21 years old (*SD* = 3.96 years), and 39% (*n* = 25) identified as Asian or Asian American, 59% (*n* = 38) identified as women, and 78% (*n* = 50) reported having no experience or familiarity with brain stimulation. To partake in our experiment, participants must have been between 18 and 35 years old and have no metallic implants in the head; no implanted internal or external electrical stimulation devices; no previous history of seizures, head injuries, or other brain-related conditions; no previous or current illnesses that have caused brain injury; no orthopedic injuries or problems with the lower back, hip, knees, or ankles; no stretched earlobes or gauges; no a sensitive scalp, the ability to remove ear piercings and jewelry; and could not have been pregnant or nursing.

### 2.3. Materials and Equipment

#### 2.3.1. CES Device

The Alpha-Stim^®^ M (Electromedical Products International Inc., Mineral Wells, TX, USA) is cleared for distribution by the Food and Drug Administration (K903014, K896948) to treat anxiety, insomnia, depression, and other clinical disorders. The device delivers pulsing current stimulation through two earlobe electrode clips at customizable current intensities, frequencies, and durations. We applied 100 μA at 0.5 Hz for a maximum of 20 min. A total of 100 μA at 0.5 Hz is typical for CES study procedures, though durations can range from 20 to 60 min [22,38,43,44,45,46,47,48,49,50]. As performed in previous research [44], we used two electrode cables, one intact (active) and one modified (sham). To produce the modified sham cable, we electrically shorted the anodal and cathodal leads in the middle of the otherwise intact cable, causing the device to detect low impedances and behave identically for both active and sham conditions; critically, however, no current reached the electrodes when using the modified sham cable. Researchers (aside from the principal investigator) and participants could not decipher which cable was active versus sham, at least not based on physical appearance or behavior.

#### 2.3.2. Survey Measures

Self-report measures relevant to the current study included vestibular sensations and cutaneous sensations. Demographic information was also collected [51]. Several other questionnaires were administered via a tablet and are intended to be used for various exploratory analyses. For conciseness, these questionnaires are not reported in this manuscript.

Vestibular Sensations. Vestibular sensations assessed participants’ subjective experiences of balance across six areas: dizziness, unsteadiness, lightheadedness, fatigue, false sense of motion or spinning sensations, and difficulty concentrating. Items were selected from a suggested algorithm for diagnosing dizziness [52]. Participants had five options to rate the severity of each sensation: “not at all” (0), “slightly” (1), “moderately” (2), “very” (3), or “extremely” (4). The original intention was to average items, but modifications were made based on the item distributions as described in the Results section. Vestibular sensations were assessed a total of nine times: at baseline, as well as after each trial. Participants could optionally provide open-ended responses for any additional vestibular sensations they experienced.

Cutaneous Sensations. Cutaneous sensations from the CES device were assessed at the end of the fourth and eighth trials across five areas: distraction, warmth or hotness, pain, itchiness, and discomfort [32]. Participants had five answer options to rate the severity of each sensation: “extremely mild” (0), “mild” (1), “moderate” (2), “severe” (3), or “extremely severe” (4). Participants were asked if they believed they received active or sham stimulation; the three possible response options were “active”, “sham”, and “I don’t know”. If participants selected either “active” or “sham”, they indicated confidence in their answer using a slider scale ranging from “not at all confident” (0) to “extremely confident” (100). Participants could optionally provide open-ended responses for any additional cutaneous sensations they experienced.

#### 2.3.3. Wearable Balance Sensors

APDM Opal (APDM Wearable Technologies Inc., Portland, OR, USA) wearable synchronized sensors are inertial measurement units (IMUs). Participants wore two sensors attached to adjustable straps. One sensor was strapped on the sternum and measured static postural sway data. The measure of interest for sway data was mean root mean square (RMS) sway (m/s^2^), which indicates steadiness (i.e., center of pressure, variability, and displacement). The other sensor was strapped on the lower lumbar (L5-S1) and captured the dynamic change of transitional sit-to-stand (STS) movements. The measure of interest for STS data was the mean lean angle across the 30 s trial in degrees (°). During the study, participants placed the edges of their feet alongside a standardized footplate. Data were streamed in real-time to a tablet and were hosted on Mobility Lab, a portable gait and balance system that provides sensitive, valid, and reliable outcome measures [53].

#### 2.3.4. Serial Subtraction Task

The serial subtraction task was verbalized serial subtractions of seven from randomly selected three-digit numbers [54]. This procedure was inspired by other studies conducted by our research group [55], as well as other researchers [33,37]. The baseline number for the serial subtraction task was 885. The subsequent numbers were 692, 241, 598, and 224. All participants received the numbers in the same temporal order to ensure consistency, though the timing of this experimental condition varied across participants, given the counterbalanced design.

### 2.4. Procedure

After receiving a study overview, participants completed baseline questionnaires that measured initial subjective vestibular sensations. After, the researcher used visual aids to explain how to perform the static sway and dynamic STS positions. The researcher explained that during each trial, the participant would remain quiet or verbally perform the serial subtraction task. Tones signaled the beginning and ending of each trial.

Next, participants put on the sternum and lumbar sensors attached to adjustable straps. After participants completed a baseline of the serial subtraction task, the researcher selected the randomized cable for the first four trials. Participants secured an adjustable strap around their abdomen, and the researcher clipped the CES device onto the strap. The device was turned on with the appropriate settings, and the participant put on the ear clips.

Participants completed eight trials, with each trial lasting 30 s, in a single testing session. After each trial, participants reported their vestibular sensations. Between the fourth and fifth trials, the cables were switched. During this switch, participants completed an additional questionnaire that assessed cutaneous sensations. These same surveys were administered at the end of the eighth trial. Once the eight trials were completed, participants removed the equipment and completed a final round of questionnaires. Finally, participants were thanked, debriefed, and compensated.

### 2.5. Analyses

We used R (v4.1.3) [56] for data processing (*tidyverse* v1.3.2 [57]), preliminary data analysis (*psych* v2.2.9 [58]; *jmv* v2.3.4 [59]; *epiDisplay* v3.5.0.2 [60]), and primary data analysis (*lmerTest* v3.1-3 [61]; *performance* v0.10.4, [62]).

We used multilevel modeling (MLM) to determine if differences in the frequency of self-reported vestibular sensations and performance-based balance metrics varied within and across participants as explained by stimulation, physical demand, and cognitive demand. For each outcome, we first evaluated the need for MLM by partitioning the variance with null models that contained no predictors. These null models specified a random effect of the intercept. We quantified the variance in the outcome variable and partitioned this variance into between- and within-participant levels by calculating the intraclass correlation (ICC).

Predictors were individually added to each model to estimate how much variance each variable could explain across each of the three outcomes. The first step for each model specified trial as a fixed effect to determine whether order effects needed to be considered to determine the influence of the subsequent predictors. The second step of model estimation specified stimulation as a fixed effect (reference condition: sham). Next, when applicable, we included physical demand as a fixed effect (reference condition: static sway). After, we added cognitive demand as a fixed effect (reference condition: low demand/remain silent). Finally, we specified any possible two- and three-way interactions as fixed effects. The partial and full models included a random effect of the intercept.

For the preliminary analysis, we examined the descriptive statistics for all variables. For the primary analysis, all models were fit using maximum likelihood. We used likelihood ratio tests to compare the goodness of fit across models. No outliers were detected when we examined post-analysis diagnostic plots for homogeneity, normality, and independence.

## 3. Results

### 3.1. Preliminary Analysis

#### 3.1.1. Self-Reported Vestibular and Cutaneous Sensations

Initially, we intended to average the six vestibular sensation items as a measure of symptom severity ranging from “not at all” (0) to “extremely” (4). However, the skewness (ranges: 1.01–3.16) and kurtosis (ranges: 3.75–13.22) values for all items were high, indicating non-normal distributions [63]. Scores were re-coded as binaries to account for these non-normal distributions. Scores of 0 were retained to indicate the absence of sensation, and scores ≥ 1 were re-coded as 1 to indicate the presence of any sensations. These six binary variables were summed to represent the total frequency of reported vestibular sensations pooled from all six items, which ranged from 0 (“experienced no sensations”) to 6 (“experienced all sensations”). The average number of reported vestibular sensations was 1.70 (*SD* = 1.70), with an approximately symmetric (skewness = −0.99) and relatively mesokurtic (kurtosis = 3.16) distribution (see Table 1).

Participants reported whether they believed they received active or sham stimulation after the fourth and eighth trials (see Table 2). The relationship between the stimulation condition and participant identification for the stimulation condition was not significant (χ^2^(1, 91) = 2.55, *p* = .110), meaning our blinding procedure was effective.

Cutaneous sensations were reported to be extremely mild (i.e., ratings of 0) for distraction, warmth or hotness, pain, itchiness, and discomfort. The item distributions were right-skewed (skewness range: 1.04–5.31) and heavy-tailed (kurtosis range: 3.43–33.61). Per paired samples *t*-tests, no significant differences (*p*s ≥ .058) existed between active and sham stimulation across severity ratings of the five cutaneous sensations. These results suggest that any observed stimulation effects can confidently be attributed to CES application.

#### 3.1.2. Balance Performance

**Sway.** Across 255 sway trials, the mean RMS sway was 0.12 m/s^2^ (*SD* = 0.13 m/s^2^, range = 0.02–1.21 m/s^2^). Data from one trial were missing due to a software error. The distribution was right-skewed (skewness = 5.51) and heavy-tailed (kurtosis = 41.79). Further examination showed that the mean RMS sway in the single-task condition was 0.08 m/s^2^ (*SD* = 0.04 m/s^2^, range = 0.03–0.36 m/s^2^). In the dual-task condition, the mean RMS sway was 0.17 m/s^2^ (*SD* = 0.17 m/s^2^, range = 0.02–1.21 m/s^2^). Both distributions for RMS sway by cognitive demand were right-skewed (single-task skewness = 2.40, dual-task skewness = 4.25) and heavy-tailed (single-task kurtosis = 12.90, dual-task skewness = 24.42).

**Sit-to-Stand (STS).** Across 256 STS trials, the mean lean angle was 20.49° (*SD* = 7.98°, range = 9.49–57.61°). The distribution was slightly right-skewed (skewness = 1.69) and heavy-tailed (kurtosis = 7.16).

### 3.2. Primary Analysis

#### 3.2.1. Self-Reported Vestibular Sensations

Around 51% of the variance in the number of reported vestibular sensations was between participants. Only two models showed significantly improved model fit compared to the null model (see Table 3). For conciseness, we report the parameter results from Model 3, which specified trial, stimulation, and physical demand as the fixed effects and the intercept as a random effect (see Table 4).

Participants who received sham stimulation while standing during their first trial were predicted to report approximately one vestibular sensation; this estimate significantly differed from zero (γ_00_ = 1.28, *p* ≤ .001, 95% CI [0.91, 1.66]). The intercept varied across all participants (*τ*_00_ = 1.51, 95% CI [1.02, 1.50]). The fixed effect for trial was significant, indicating the number of reported vestibular sensations slightly increased over the course of the multiple trials (γ_10_ = 0.08, *p* ≤ .001, 95% CI [0.03, 0.12]). The fixed effect for stimulation was not significant, meaning there were no differences in the number of reported sensations as explained by stimulation after accounting for the trial number (γ_20_ = 0.09, *p* = .408, 95% CI [−0.12, 0.29]). Further, the fixed effect for physical demand was significant, meaning participants reported slightly more vestibular sensations when performing the dynamic STS than the static sway, holding all other variables constant (γ_30_ = 0.23, *p* = .026, 95% CI [0.03, 0.43]).

#### 3.2.2. Balance Performance

**Sway.** Approximately 1% of the variance for RMS sway was between participants, meaning the remaining 99% of the variation was within participants. Only Model 3, which included trial, stimulation, and cognitive demand as fixed effects and the intercept as a random effect, showed a significant improvement in model fit compared to the null model (see Table 5 for model comparisons and Table 6 for parameter estimates).

Participants who remained quiet while receiving sham stimulation were predicted to have an average RMS sway of 0.10 m/s^2^ during their first trial; this estimate significantly differed from zero (γ_00_ = 0.10, *p ≤* .001, 95% CI [0.07, 0.35]). The random effect of the intercept implied that this estimate did not significantly vary across participants (*τ*_00_ = 0.00, 95% CI [0.00, 0.05]). A significant fixed effect was observed for the trial, where postural steadiness decreased by 0.01 m/s^2^ throughout multiple trials (γ_10_ = −0.01, *p* = .040, 95% CI [−0.03, 0.00]). The fixed effect for stimulation was not significant, demonstrating no differences in postural steadiness between active and sham after accounting for trial number (γ_20_ = 0.01, *p* = .539, 95% CI [−0.02, 0.04]). There was a significant fixed effect for cognitive demand, where participants were more unsteady when counting backward compared to when they remained silent while holding other variables constant (γ_30_ = 0.08, *p* ≤ .001, 95% CI [0.05, 0.11]).

**Sit-to-Stand (STS).** Approximately 74% of the variance for STS mean lean angle was between participants. Three of the four models significantly improved model fit compared to the null model (see Table 7). For conciseness, we report the results from Model 3, which specified trial, stimulation, and cognitive demand as fixed effects and the intercept as a random effect (see Table 8).

Participants who remained quiet while receiving sham stimulation for their first trial were predicted to have an average lean angle of 19.98°; this intercept estimate was significantly different than zero (γ_00_ = 19.98, *p* ≤ .001, 95% CI [18.00, 21.97]). The intercept significantly varied across all participants (*τ*_00_ = 47.42, 95% CI [5.76, 8.37]). The fixed effect for trial was significant, indicating the lean angle decreased by −0.60° as the number of trials increased (γ_10_= −0.60, *p* = .007, 95% CI [−1.01, −0.16]). The fixed effect for stimulation was also significant; the slope for mean lean angle increased by 1.34° when participants received active stimulation compared to sham (γ_20_ = 1.34, *p* = .006, 95% CI [0.39, 2.29]). The fixed effect for cognitive demand was also significant, where the slope of the mean lean angle was higher by 1.43° when participants counted backward as opposed to remaining quiet, holding all other variables constant (γ_30_ = 1.43, *p* = .003, 95% CI [0.48, 2.38]).

## 4. Discussion

The current study investigated whether physical and cognitive demand manipulations can influence the frequency and intensity of CES side effects, as measured via survey and sensor metrics, in a non-clinical sample. Several results are relevant to the study’s aim. Firstly, participants did not report more vestibular sensations when receiving active stimulation than with sham. The likelihood of reporting such sensations increased slightly when the physical demand was high. Additionally, we observed that high cognitive demand significantly increased postural sway while stimulation did not. Lastly, we noticed minor positive effects of active stimulation on balance during dynamic situations with high physical demand.

Collectively, our findings suggest that CES is unlikely to induce vestibular side effects unless it is for a specific outcome under certain circumstances. Our results align with the previous research that CES does not cause serious side effects [5,6], and we contributed evidence that expands upon this claim by manipulating users’ physical and cognitive capacity. We also measured vestibular sensations in real-time using survey and sensor outcomes rather than simply asking about side effects at the end of the study protocol, making it easier to parse whether the CES device contributed to the side effects.

Regarding the first finding, we hypothesized that participants would report more vestibular sensations when they received active rather than sham stimulation. We may have observed null effects of stimulation for reported vestibular sensations because our questionnaire was not a sensitive enough measure. Existing scales that evaluate vestibular sensations, such as the Dizziness Handicap Inventory [64], are intended to diagnose clinical populations experiencing persistent symptoms [65] and may not readily apply to our participants who are healthy young adults and are experiencing relatively transient side effects. Relatedly, the severity of side effects may differ between clinical and non-clinical samples, where common symptoms for a clinical health condition may intensify when applying CES [6].

A ramp-up-to-threshold procedure for the stimulation settings could help determine the sensitivity of vestibular sensations. Rather than having a fixed current level, future studies should allow participants to slowly ramp up the stimulation level until they start to experience vestibular sensations. At this point, participants’ thresholds could be set just below this inflection point. Such an approach could account for individual differences in sensitivity that are masked using a fixed stimulation level for all participants. Comparisons of these inflection points could be made across different samples to determine the generalizability of our conclusions.

For the second finding, stimulation may have a significant effect during dynamic STS, given the placement location of the earlobe electrode clips. Behind the ears are the mastoids, where the vestibular nerve runs from the inner ear towards the vestibular brain stem nuclei, which connect to thalamic relay stations that integrate and relay sensory signals to higher brain regions [66,67]. As previously mentioned, GVS stimulates the vestibular system via the mastoids at ≤1 mA, which sends a sensory signal that deceives the body into perceiving an unplanned physical movement in space as though it originated from the body’s own movement [68,69,70]. Noisy GVS (nGVS) has been found to alter the postural sway of healthy young adults [71]. More investigation is needed into the similarities and differences between CES and (n)GVS.

Another potential direction for future research is to diversify physical demands to include tasks like the stand and walk (SAW) test and the timed up and go (TUG) test, both of which can be administered with APDM Opal (APDM Wearable Technologies Inc.) IMUs. These tasks are more complex and thus could help us further explore whether the effect of stimulation generalizes beyond the STS. In addition, multiple iterations of the same condition for a longer duration would be beneficial to determine the consistency of our results. In addition to these design modifications, a promising analysis technique would be analyzing sub-second time periods to quantify the on-set and off-set balance issues arising from stimulation and other possible physical and cognitive response conflicts [72].

## 5. Conclusions

The reliability and generalizability of our results must be cemented by future research that addresses the current study’s limitations. Nevertheless, we have provided preliminary evidence that CES does not induce vestibular sensations, even when physical and cognitive demands are present. Instead, dynamic physical demand is more likely to explain reports of elevated vestibular sensations, and high cognitive demand can better explain sway imbalance for static situations. In dynamic physical situations, however, stimulation can significantly predict imbalance. Identifying and quantifying any potential risks and consequences of CES is necessary for optimizing the potential benefits. The results of this study must be considered when deliberating how to guarantee the safety of and maximize performance with CES devices.

## Figures and Tables

**Table 1:** Number of vestibular sensations reported across all trials.

Number of Reported Sensations	*n* Trials (%)
Zero (none)	153 (30%)
One	131 (26%)
Two	94 (18%)
Three	56 (11%)
Four	30 (6%)
Five	23 (4%)
Six (all)	25 (5%)

Note. *N* = 512 trials.

**Table 2:** Participant identification of stimulation condition.

Possible Guess	*n* Guesses (%)	% Correct Guesses
Active	34 (27%)	62%
Sham	57 (44%)	58%
Unsure	37 (29%)	-

Note. *N* = 128 guesses.

**Table 3:** Comparisons across all models estimating the frequency of self-reported vestibular sensations.

Model (M)		−2Loglikelihood (No. Parameters)	Deviance (Δ Previous M)	χ^2^ Critical Value (Δ df)	*R* ^2^	*p*	Conclusion
No.	Predictors						
0	None (null)	−883.82 (3)	1767.6		.52		
1	Trial	−877.51 (4)	1755.0 (12.6)	12.63 (1)	.53	≤.001 ***	Improved model fit
2	Trial, stimulation	−877.18 (5)	1754.3 (0.7)	0.66 (1)	.53	.418	Unimproved model fit
**3**	**Trial, stimulation, physical demand**	**−874.71 (6)**	**1749.4 (4.9)**	**4.94 (1)**	**.54**	.026 *	**Improved model fit**
4	Trial, stimulation, physical demand, cognitive demand	−874.62 (7)	1749.2 (0.2)	0.18 (1)	.54	.676	Unimproved model fit
5	M4 with stimulation × physical demand	−874.20 (8)	1748.4 (0.8)	0.84 (1)	.54	.359	Unimproved model fit
6	M5 with stimulation × cognitive demand	−873.80 (9)	1747.6 (0.8)	0.80 (1)	.54	.371	Unimproved model fit
7	M6 with physical demand × cognitive demand	−871.95 (10)	1743.9 (3.7)	3.70 (1)	.54	.054	Unimproved model fit
8	Full (M7 with stimulation × physical demand × cognitive demand)	−871.47 (11)	1743.0 (0.9)	0.95 (1)	.54	.329	Unimproved model fit

Note. The bolded row indicates the model presented in the main text. *** *p* ≤ .001, * *p* ≤ .05.

**Table 4:** Parameter estimates of the best fitting model estimating the frequency of self-reported vestibular sensations.

**Fixed Effects**							
**Parameter**		**Estimate**	***SE***	**df**	***t***	***p***	**95% CI [LL, UL]**
γ_00_	Intercept	1.28	0.19	120.37	6.73	≤.001 ***	[0.91, 1.66]
γ_10_	Trial	0.08	0.02	448	3.36	≤.001 ***	[0.03, 0.12]
γ_20_	Stimulation	0.09	0.10	448	0.83	.408	[−0.12, 0.29]
γ_30_	Physical demand	0.23	0.10	448	2.23	.026 *	[0.03, 0.43]
**Random effects**							
**Parameter**		**Variance**	***SD***	**95% CI [LL, UL]**			
*τ*_00_	Intercept	1.51	1.23	[1.02, 1.50]			
*σ* ^2^	Residual	1.34	1.16	[1.08, 1.24]			

Note. Stimulation levels: 0 = sham CES, 1 = active CES. Physical demand levels: 0 = static sway, 1 = dynamic sit-to-stand (STS). *** *p* ≤ .001, * *p* ≤ .05.

**Table 5:** Comparisons across all models estimating RMS sway.

Model (M)		−2Loglikelihood (No. Parameters)	Deviance (Δ Previous M)	χ^2^ Critical Value (Δ df)	*R* ^2^	*p*	Conclusion
No.	Predictors						
0	None (null)	152.87 (3)	−305.74		.01		
1	Trial	154.77 (4)	−309.54 (3.8)	3.80 (1)	.03	.051	Unimproved model fit
2	Trial, stimulation	154.93 (5)	−309.86 (0.32)	0.33 (1)	.03	.567	Unimproved model fit
**3**	**Trial, stimulation,** **cognitive demand**	**167.93 (6)**	**−335.87 (26.01)**	**26.00 (1)**	**.16**	≤.001 ***	**Improved model fit**
4	M3 with stimulation × cognitive demand	167.93 (7)	−335.87 (0)	0.00 (1)	.16	.958	Unimproved model fit

Note. The bolded row indicates the model presented in the main text. *** *p* ≤ .001.

**Table 6:** Parameter estimates of the best fitting model estimating RMS sway.

**Fixed Effects**							
**Parameter**		**Estimate**	***SE***	**df**	***t***	***p***	**95% CI [LL, UL]**
γ_00_	Intercept	0.10	0.02	254.89	6.09	≤.001 ***	[0.07, 0.35]
γ_10_	Trial	−0.01	0.01	191.65	−2.07	.040 *	[−0.03, 0.00]
γ_20_	Stimulation	0.01	0.02	191.40	0.62	.539	[−0.02, 0.04]
γ_30_	Cognitive demand	0.08	0.02	192.72	5.28	≤.001 ***	[0.05, 0.11]
**Random effects**							
**Parameter**		**Variance**	***SD***	**95% CI [LL, UL]**			
*τ*_00_	Intercept	0.00	0.03	[0.00, 0.05]			
*σ* ^2^	Residual	0.01	0.01	[0.11, 0.14]			

Note. Measurement metric is m/s^2^. Stimulation levels: 0 = sham CES, 1 = active CES. Cognitive demand levels: 0 = remain quiet/single-task, 1 = count backward/dual-task. *** *p* ≤ .001, * *p* ≤ .05.

**Table 7:** Comparisons across all models estimating sit-to-stand (STS) mean lean angle.

Model (M)		−2Loglikelihood (No. Parameters)	Deviance (Δ Previous M)	χ^2^ Critical Value (Δ df)	*R* ^2^	*p*	Conclusion
No.	Predictors						
0	None (null)	−802.24 (3)	1604.5		.74		
1	Trial	−799.57 (4)	1599.2 (5.3)	5.34 (1)	.75	.021 *	Improved model fit
2	Trial, stimulation	−795.89 (5)	1591.8 (7.4)	7.37 (1)	.76	.007 **	Improved model fit
**3**	**Trial, stimulation,** **cognitive demand**	**−791.56 (6)**	**1583.1 (8.7)**	**8.66 (1)**	**.77**	.003 **	**Improved model fit**
4	M3 with stimulation × cognitive demand	−790.88 (7)	1581.8 (1.3)	1.37 (1)	.77	.242	Unimproved model fit

Note. The bolded row indicates the model presented in the main text. ** *p* ≤ .01, * *p* ≤ .05.

**Table 8:** Parameter estimates of the best fitting model estimating sit-to-stand (STS) mean lean angle.

**Fixed Effects**							
**Parameter**		**Estimate**	***SE***	**df**	***t***	***p***	**95% CI [LL, UL]**
γ_00_	Intercept	19.98	1.00	98.64	19.97	≤.001 ***	[18.00, 21.97]
γ_10_	Trial	−0.60	0.22	192.00	−2.72	.007 **	[−1.01, −0.16]
γ_20_	Stimulation	1.34	0.48	192.00	2.77	.006 **	[0.39, 2.29]
γ_30_	Cognitive demand	1.43	0.48	192.11	2.98	.003 **	[0.48, 2.38]
**Random effects**							
**Parameter**		**Variance**	***SD***	**95% CI [LL, UL]**			
*τ*_00_	Intercept	47.42	6.89	[5.76, 8.37]			
*σ* ^2^	Residual	14.71	3.84	[3.48, 4.25]			

Note. Measurement metric is degrees (°). Stimulation levels: 0 = sham CES, 1 = active CES. Cognitive demand levels: 0 = remain quiet/single-task, 1 = count backward/dual-task. *** *p* ≤ .001, ** *p* ≤ .01.

## Data Availability

The data presented in this study are openly available on the Open Science Framework (OSF) at https://osf.io/qw9ta/ (accessed on 15 January 2024).
